# Porcine Viperin protein inhibits the replication of classical swine fever virus (CSFV) in vitro

**DOI:** 10.1186/s12985-017-0868-4

**Published:** 2017-10-23

**Authors:** Wenliang Li, Li Mao, Yongguo Cao, Bin Zhou, Leilei Yang, Linxiao Han, Fei Hao, Tao Lin, Wenwen Zhang, Jieyuan Jiang

**Affiliations:** 1 Institute of Veterinary Medicine, Jiangsu Academy of Agricultural Sciences, Key Laboratory of Veterinary Biological Engineering and Technology, Ministry of Agriculture, Nanjing, 210014 China; 2 College of Veterinary Medicine, Jilin University, Changchun, 130062 China; 3 College of Veterinary Medicine, Nanjing Agricultural University, Nanjing, 210095 China; 4 Department of Chemistry and Biochemistry, South Dakota State University, Brookings, SD 57007 USA

**Keywords:** Viperin, CSFV, Antiviral, Co-localization, Interaction

## Abstract

**Background:**

*Classical swine fever virus* (CSFV) is the causative pathogen of Classical swine fever (CSF), a highly contagious disease of swine. Viperin is one of the hundreds of interferon-stimulated genes (ISGs), and possesses a wide range of antiviral activities. The aim of this study was to explore whether porcine Viperin has the anti-CSFV activity.

**Method:**

The influences of CSFV infection on Viperin expression and *Newcastle disease virus* (NDV)/*Pseudorabies virus* (PRV)-induced Viperin expression were examined in 3D4/21 cells and porcine peripheral blood mononuclear cells (PBMCs). Porcine Viperin gene was amplified to generate cell line PK-Vi over-expressing Viperin. CSFV was inoculated in the cell lines and viral load was detected by qRT-PCR, virus titration and Western blot. The influence of Viperin expression on CSFV binding, entry and release in the cells was also examined. The co-localization of Viperin with CSFV and its proteins (E2, NS5B) was determined by confocal laser scanning microscopy test. Co-IP assay was performed to check the interaction of Viperin with CSFV proteins.

**Results:**

CSFV infection could not induce Viperin expression in vitro while significantly inhibiting NDV/PRV-induced Viperin expression at 12, 24 and 48 h post infection (hpi; *P* < 0.05). The proliferation of CSFV in PK-Vi was significantly inhibited at 24, 48 and 72 hpi (P < 0.05), comparing with control cells (PK-C1 expressing EGFP). Virus in both cell culture supernatants and cell pellets were reduced equally. CSFV binding and entry in the cells were not interfered by Viperin expression. These results indicated its anti-CSFV function occurred during the genome and/or protein synthesis step. Confocal laser scanning microscopy test showed the Viperin-EGFP protein co-localized with CSFV E2 protein in CSFV infected PK-Vi cells. Further experiments indicated that Viperin protein co-localized with E2 and NS5B proteins of CSFV in the transfected 293 T cells. Furthermore, Co-IP assay confirmed the interaction of Viperin with E2 protein, but not NS5B.

**Conclusion:**

Porcine Viperin effectively inhibited CSFV replication in vitro, potentially via the interaction of Viperin with CSFV E2 protein in cytoplasm. The results provided foundation for further studies of the interaction of Viperin with CSFV and other viruses.

## Background

Classical swine fever (CSF) is a highly contagious disease, and has severe impact on the swine industry worldwide [1]. The causative agent, *Classical swine fever virus* (CSFV), belongs to the genus *pestivirus* of the *Flaviviridae* family, along with *Bovine viral diarrhea virus 1* and *2* (BVDV 1, BVDV 2), *Border disease virus* (BDV) and several newly found atypical *pestiviruses* [2, 3]. In China, CSF is still one of the most important infectious diseases and the Hog cholera lapinized virus (HCLV) vaccine has been widely used to prevent and control the disease [4].

The first line of defense against viral infection is the innate immunity, especially the type I interferon (IFN) response, which consequently triggers the expression of hundreds of interferon-stimulated genes (ISGs), such as protein kinase R (PKR), the GTPase Mx1, ISG15, IFIT and so on [5, 6]. Viperin (*v*irus *i*nhibitory *p*rotein, *e*ndoplasmic *r*eticulum-associated, *in*terferon-inducible) is one of the few ISGs shown to have direct antiviral activity to a broad range of viruses and modulating innate immune signaling [7]. Viperin, also known as cytomegalovirus-induced gene (cig 5), was first identified as an induced gene in fibroblasts infected with *Human cytomegalovirus* (HCMV) [8]. Since then, Viperin has been found in a wide range of species [9]. Over the last several years, Viperin showed antiviral activity against a range of DNA and RNA viruses, including HCMV, HCV, *West Nile virus* (WNV), *Dengue virus*, *Influenza A virus*, VSV, HIV-1, *Equine infectious anemia virus*, *Respiratory syncytial virus* and so on [8, 10–16]. Porcine Viperin gene has been identified but no report about its antiviral function was available.

Viruses have evolved many strategies to counteract host immune responses. Studies have been performed on the effect of CSFV infection on host immune responses and antiviral genes expression [17, 18]; and CSFV has been confirmed to inhibit type I IFN response (IFN-α/β induction) by direct or indirect interaction of N^pro^ with interferon regulatory factor 3 (IRF3) and IRF7 [19, 20]. Human MxA, porcine Mx1 and GBP1 have been confirmed to suppress CSFV replication in vitro [21, 22]. The effect of CSFV infection on Viperin expression and anti-CSFV activity of Viperin has not been reported. In this study, we examined the effect of CSFV infection on Viperin expression or NDV/PRV-induced Viperin expression in porcine alveolar macrophage cell line 3D4/21 and porcine peripheral blood mononuclear cells (PBMCs). The anti-CSFV activity of Viperin was determined on cell line PK-Vi, which stably expressed the EGFP-Viperin fusion protein. Moreover, the mechanism of its anti-CSFV activity was explored.

## Methods

### Cells and virus

PK-15, 3D4/21 and 293 T cells were propagated in DMEM (Hyclone, USA) supplemented with 10% fetal bovine serum (Gibco, USA), 100 μg/ml streptomycin and 100 IU/ml penicillin. Porcine PBMCs were separated by gradient centrifugation from the peripheral blood of a healthy pig and re-suspended (5 × 10^5^ cells/ml) in RPMI-1640 medium (Hyclone, USA) supplemented with 10% fetal bovine serum (Gibco, USA), 100 μg/ml streptomycin and 100 IU/ml penicillin. The virulent CSFV Shimen strain was obtained from the National Institute of Veterinary Drug Control of China and tittered on PK-15 cells. NDV (Lasota strain) and PRV (Bartha K-61) were obtained from Tech-Bank Bio-tech cooperation Ltd. (Nanjing, China).

### Construction of the eukaryotic expression plasmids

PBMCs (5 × 10^5^ cells/ml) were seeded on 6-well plate and stimulated by concanavalin A (ConA, 5 μg/ml, Sigma-Aldrich, USA). RNA was extracted from simulated PBMCs with TransZol UP reagent (Transgen, Bio, Inc., China). The Viperin coding region was amplified by a nested RT-PCR. The first RT-PCR step was carried out with Easyscript one-step RT-PCR supermix (Transgen, Bio, Inc., China) in a 20 μl reaction mixture containing 2 × R-Mix buffer, 20 pM of each primer (VF and VR, Table 1), 0.5 μl of E-Mix and 4 μl extracted RNA. Amplification products were then subjected to a second PCR step, using a pair of primers (VF1 and VR1, Table 1). PCR products were purified, digested and cloned into pEGFP-C1 at *Bgl* II and *Sal* I sites.

**Table 1:** Primers used in this study

Primer name	Sequence
VF	5′-GCTGCCATGTGGACACTGGTAC-3′
VR	5′-ATCCAGTCCCGGTCTGGTCC-3′
VF1	5′-GATAGATCTATGTGGACACTGGTAC-3’
VR1	5′-ATTGTCGACTCACCAGTCCAGCTTCAGGTCC-3’
VF2	5′-ATAAAGCTTCGCCACCATGTGGACACTGGTAC-3’
VR2	5′-ATTCTCGAGTCAAGCGTAATCTGGAACATCGTATGGGTACCAGTCCAGCTTCA-3’
qE2F	5′- GCTCCCTGGGTGGTCTAAGTC-3’
qE2R	5′- GGCTTCTGCT CACGTCGAA-3’
qViF	5′-AAGCAGAGCAGTTTGTTATCAGC-3’
qViR	5′-TTCCGCCCGTTTCTACAGT-3’
actin qF	5′-TCTGGCACCACACCTTCT-3’
actin qR	5′-TGATCTGGGTCATCTTCTCAC-3’

CSFV E2 and NS5B genes with flag tag at the 3′-end were codon optimizied, synthesized and cloned into pCMV vector to generate recombinant plasmid pCMV-E2 and pCMV-NS5B. Viperin gene with HA tag at 3′-end was amplified (VF2 and VR2, Table 1) and cloned into pcDNA3.1 to generate pcDNA-Vi. All plasmids were extracted by AxyPrep™ Plasmid Miniprep kit (Axygen, Hangzhou, China) and the concentration was measured by NanoDrop 2000 (Thermo).

### Generation of stable Viperin expressing cell lines

PK-15 cells with 80% confluence in 24-well plate were transfected with pEGFP-Vi using Lipofectamine 2000 (Invitrogen, USA) according to the manufacturer’s instructions. After twenty-four hours, fresh DMEM culture medium containing 550 μg/ml G418 (Sigma-Aldrich, USA) was added. The medium was changed every 3–5 days until G418-resistant cell foci appeared. The positive expressing cells were separated, cultivated and amplified in DMEM culture medium containing 200 μg/ml G418. The expression of target protein was confirmed by fluorescence microscopy and Western blot analysis. The resulting cell line was named as PK-Vi. The control cell line PK-C1 expressing EGFP was constructed by the same procedure using pEGFP-C1 transfected PK-15 cells. Cell viability was determined by MTT assay as done by previous reports [22, 23]. Briefly, PK-Vi, PK-C1 and PK-15 cells were seeded into 96-well plate and incubated for 5 days. 50 μl of MTT (2 mg/ml, Sigma-Aldrich) solution was added to each well and the plate was incubated at 37 °C for 4 h. After adding dimethyl sulfoxide (Sigma-Aldrich), the values of OD_560_ were detected by ELx800 (Bio-Tek). The results were expressed relative to the optical density of wells containing PK-15 cells, defined as 100% viability.

### Western blot

Cell pellets were re-suspended in PBS (with a concentration of 2 μg/μl), combined with 5 × Loading buffer, boiled and separated by 12% SDS-PAGE and transferred onto nitrocellulose membranes (Pall) using a semi-dry transfer cell (Bio-Rad) at 1 V/cm^2^ for 30 min. The membrane was treated sequentially with 1% BSA in PBST (PBS containing 0.05% Tween-20) at 37 °C for 2 h, with different primary antibodies (1/200 diluted rabbit anti-Viperin polyclonal antibody (Abcam, USA), 1/500 diluted rabbit anti-N^pro^ polyclonal antibody (kindly provided by Prof. Huaji Qiu), 1/1000 diluted rabbit anti-GFP/HA/flag antibody or 1/1000 diluted anti-β-actin monoclonal antibody (Transgen, Bio, Inc., China)) at 37 °C for 2 h, and with different secondary antibodies (rabbit anti-mouse or goat anti-rabbit IgG antibody conjugated to HRP (Transgen, Bio, Inc., China)). After three washes with PBST, the color development was performed using DAB reagents (BOSTER, Wuhan, China) or enhanced chemiluminescence luminal reagent (Thermo Scientific Pierce).

### CSFV replication detection on PK-15 cell lines

PK-C1 and PK-Vi cells were seeded in 12-well plate (10^5^ cells/ml) and infected with CSFV Shimen strain (MOI = 0.05). At 12, 24, 48 and 72 h post infection (hpi), total cell cultures, cell culture supernatants and cells pellets were collected separately. The replication dynamics of CSFV in these two cell lines and different cell compartment were determined by qRT-PCR and/or virus titration.

### Virus binding and entry detection

Cells were infected with CSFV (MOI = 1) for 1 h on ice to allow attachment but impede virus entry. After washing with ice-cold PBS, RNA was extracted for qRT-PCR to measure the amount of cell-bound virus. To test the virus entry step, the virus inoculum was removed after 1 h of binding on ice, and then cells were washed with ice-cold PBS and incubated in culture medium for 2 h at 37 °C. Cells were washed with PBS, trypsinated for 10 min, and washed again before RNA extraction and qRT-PCR detection.

### qRT-PCR

Total RNA in the samples was extracted by Transzol UP reagent (Transgen, Bio, Inc., China). The expression of Viperin mRNA or CSFV genome was identified by relative qRT-PCR, using β-actin as an endogenous control gene. The qRT-PCR amplification was carried out with TransScript Green one-step qRT-PCR supermix (Transgen, Bio, Inc., China) in a 20 μl reaction mixture containing 10 μl of 2 × Supermix, 20 pM of each primer (For CSFV: qE2F and qE2R; for Viperin: qViF and qViR; for β-actin: action qF and actin qR, Table 1), 0.5 μl of E-Mix, 0.4 μl of passive reference Dye and 4 μl extracted RNA. The reaction was run in ABI Step One following the manufacturer’s instruction: samples were incubated at 45 °C for 5 min firstly; then heated at 94 °C for 30 s and a two-step cycle (5 s at 94 °C, 30 s at 60 °C) was repeated for 40 cycles. Relative quantification of CSFV genome or Viperin mRNA was the target transcript in a treated group to that of untreated control group and expressed as –ΔΔCt.

### Virus titration

Quadruplicates of 10-fold serially diluted virus samples were added on PK-15 cell monolayer in 96-well culture plates and incubated at 37 °C for 72 h. The plates were then fixed for 30 min with absolute ethyl alcohol at 4 °C and subjected to immunofluorescence staining with the monoclonal antibody (mAb) WH303 (target to E2 protein, AHVLA, UK; 1:200 diluted in PBS) and FITC-conjugated goat anti-mouse IgG (BOSTER, Wuhan, China; 1:200 diluted in PBS). The fluorescence signal was observed under a fluorescence microscopy (ZEISS) and virus titers were calculated by Reed-Muench method and expressed as TCID_50_ per milliliter.

### Confocal laser scanning microscopy test

PK-Vi cultured on glass cover slips were infected with CSFV for 48 h. The cells were then fixed, permeabilized and subjected to immunofluorescence staining with WH303 (AHVLA, UK; 1:200 diluted) and Cy3-conjugated goat anti-mouse IgG (BOSTER, Wuhan, China; 1:100 diluted).

293 T cells cultured on glass cover slips were co-transfected with pcDNA-Vi and pCMV-E2 or pCMV-NS5B and incubated at 37 °C for 48 h. The cells were then fixed, permeabilized and subjected to immunofluorescence staining with anti-flag mAb (Beyotime Biotech, China; 1:1000 diluted) plus Alexa Fluor 555-labeled donkey anti-mouse IgG (Beyotime Biotech, China; 1:500 diluted) and rabbit anti-HA Ab (Beyotime Biotech, China; 1:100 diluted) plus Alexa Fluor 488-labeled goat anti-rabbit IgG (Beyotime Biotech, China; 1:500 diluted).

Cover slips were mounted on microscopes slides and examined by confocal laser scanning microscopy (PE, Ultra View VOX).

### Co-immunoprecipitation (co-IP)

The 293 T cells in 6 well plates were transfected with pcDNA-Vi and pCMV-E2 or pCMV-NS5B. At 48 hpi, cells were harvested and lysed using the cell lysis buffer (Beyotime Biotech, China). The lysates were centrifuged at 10,000×g for 5 min, the supernatants were incubated with HA or flag antibody overnight at 4 °C with rotation. After that, 50% suspension protein G Agarose (Beyotime Biotech, China) was added and cells were incubated for 3 h at 4 °C with rotation. Agarose containing protein complexes were washed 3 times with lysis buffer, re-suspended in 5 × Loading buffer, boiled and subjected to Western blot with rabbit anti-HA and anti-flag antibodies.

### Statistical analysis

All Data were obtained from three replicates and presented as mean ± S.D. The differences in the levels of virus load and gene expression levels were determined by one-way repeated measurement ANOVA. Statistical analyses were performed using SPSS v.16.

## Results

### CSFV infection did not induce Viperin expression but inhibited NDV/PRV-induced Viperin expression

**Fig. 1:**
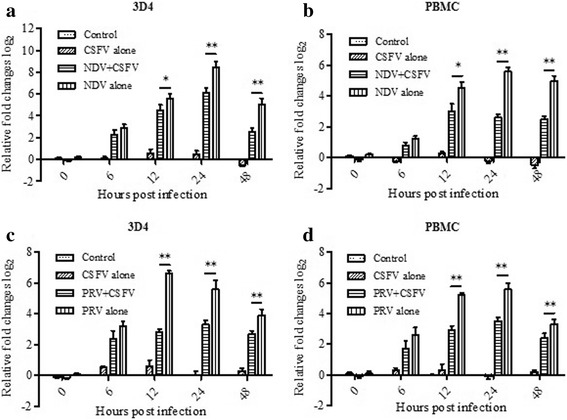
Detection of Viperin expression in 3D4/21 cells and PBMCs. **a** and **b**. 3D4/21 cells (**a**) and PBMCs (**b**) were stimulated with: 1) NDV and CSFV (MOI = 1); 2) NDV alone; 3) CSFV alone; 4) medium (negative control). **c** and **d**. 3D4/21 cells (**c**) and PBMCs (**d**) were stimulated with: 1) PRV and CSFV (MOI = 1); 2) PRV alone; 3) CSFV alone; 4) medium (negative control). Levels of Viperin mRNA presented in cell cultures of 0, 6, 12, 24 and 48 hpi were determined by relative qRT-PCR. Relative quantification of Viperin mRNA was that in treated group to that of untreated control group. The relative fold change of Viperin level was expressed as –ΔΔCt. Each treatment was performed in triplicate and data was shown as the mean ± S.D. Columns at each time point marked with * (*P* < 0.05) or ** (*P* < 0.01) are significantly different from each other

**Fig. 2:**
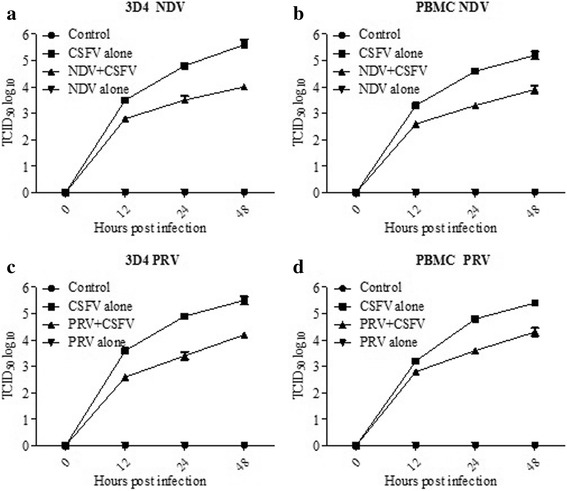
Detection of CSFV replication in 3D4/21 cells and PBMCs. CSFV titers in 3D4/21 cells (**a, c**)  and PBMCs (**b, d**) at 0, 12, 24 and 48 hpi were determined by TCID50 test. Each treatment was performed in triplicate and data was shown as the mean ± S.D.

NDV and PRV were confirmed to induce Type I IFNs and Viperin expression [24–26] and were used as a Viperin inducer in this test. 3D4/21 cells and porcine PBMCs were used to test the effect of CSFV infection on Viperin mRNA expression. Cells at 80% confluence in 12-well plates were treated with different inoculation procedures (NDV/PRV alone; CSFV alone; NDV/PRV and CSFV; MOI = 1) and the Viperin mRNA levels at 0, 6, 12, 24 and 48 hpi were calculated by qRT-PCR. As shown in Fig. 1a and c, under the stimulation of NDV and PRV, 3D4/21 cells (NDV alone; PRV alone) could produce high levels (2^2.6^–2^8.4^ times to the control groups) of Viperin mRNA expression from 6 hpi and peaked at 12 or 24 hpi, but Viperin was not significantly induced in CSFV infected cells (CSFV alone). Similar results were observed in PBMCs (Fig. 1b, d). While in the co-infected cells (NDV + CSFV; PRV + CSFV), NDV induced Viperin was significantly decreased by 2.1-, 4.9- and 5.7-fold at 12, 24 and 48 hpi, respectively (Fig. 1a). PRV induced Viperin mRNA expression was significantly decreased by 10.6-, 4.9- and 2.3-fold at 12, 24 and 48 hpi, respectively (Fig. 1c). Results in PBMCs were similar: NDV and PRV-induced Viperin were significantly decreased by 2.8-, 8-, 5.7-fold and 4.9-, 3.7-, 1.9-fold at 12, 24 and 48 hpi, respectively (Fig. 1b, d). In addition, CSFV replication levels in these cells were detected, as Fig. 2 shown, CSFV could proliferate in NDV or PRV co-infected cells but the titers were much lower (1.2–1.6 log_10_ times reduction at 24-48hpi) than those of CSFV infected cells (CSFV alone group). These results demonstrated that the CSFV Shimen strain could not induce the Viperin expression, but inhibit the induction of Viperin expression by other viruses.

### Generation and validation of cell lines

**Fig. 3:**
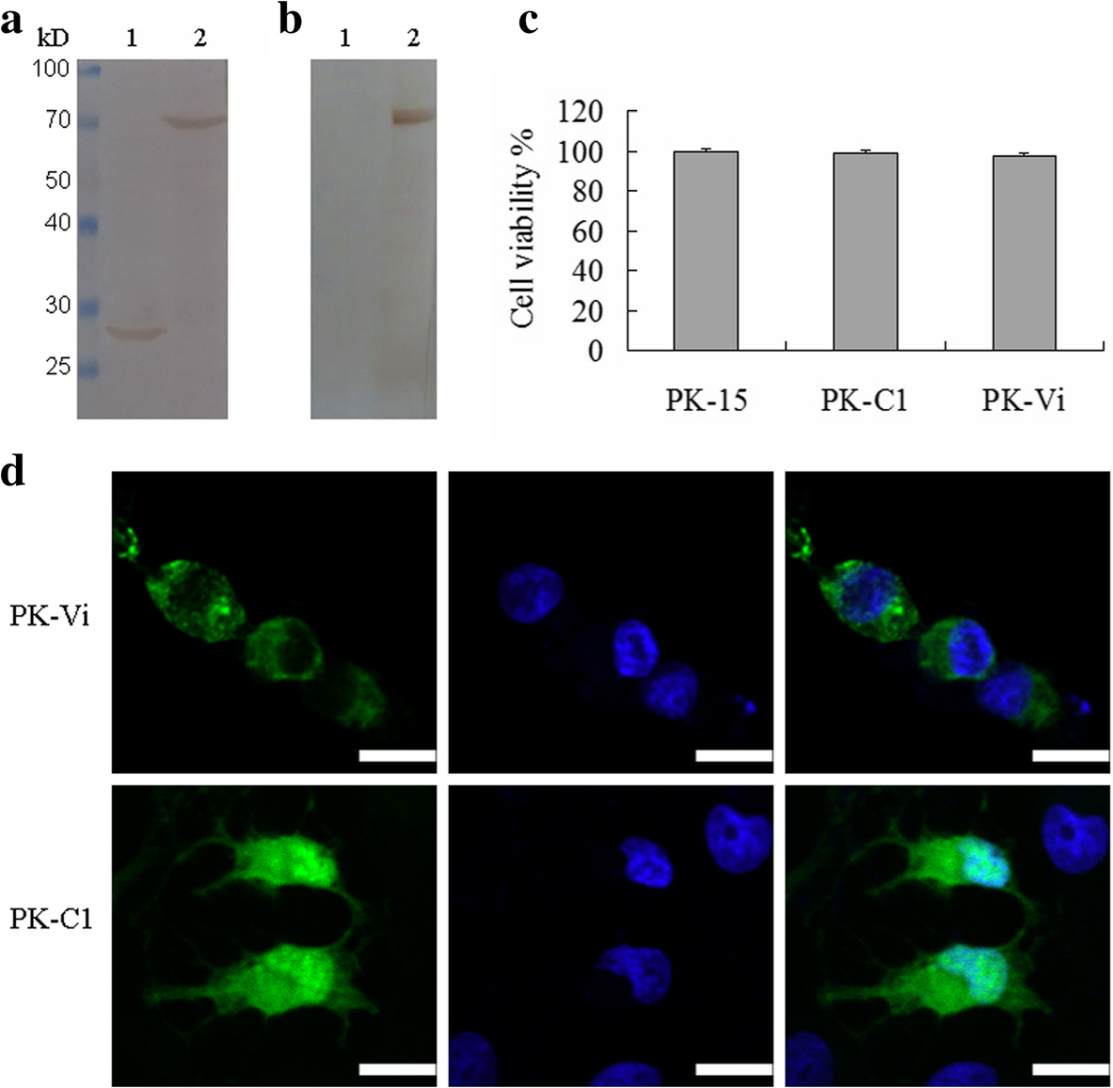
Generation and validation of stably-transfected cell lines. **a** and **b**. Western blot identification of PK-Vi and PK-C1 by anti-GFP antibody (**a**) and anti-Viperin antibody (**b**). lane 1, PK-C1; lane 2, PK-Vi. **c**. PK-Vi, PK-C1 and PK-15 cells were seeded into 96-well plate and incubated for 5 days and cell viability was determined by MTT assay. The cell viability of PK-15 was used as control (100%). Data are expressed as mean (% of control) ± S.D. **d**. Confocal microscopy examination of PK-Vi and PK-C1. Nuclei were stained with DAPI (Beyotime, Biotech, China) and the cells were examined by confocal laser scanning microscopy (PE, Ultra View VOX). Scale bar indicated 20 μm

The porcine Viperin gene was successfully amplified from ConA stimulated porcine PBMCs and cloned into pEGFP-C1 vector. The obtained Viperin gene possessed 99.9% identity with previously reported sequences in Genbank. The plasmids pEGFP-Vi and pEGFP-C1 were transfected into the PK-15 cells to get the cell lines PK-Vi and PK-C1, respectively. The EGFP-Viperin fusion protein was successfully expressed as examined by fluorescence microscopy. In Western blot detection, lysates of PK-Vi and PK-C1 cells showed protein bands with molecular weight of approximately 70 kD and 27 kD, respectively (Fig. 3a); the lysates of PK-Vi cells showed the 70 kD band reacting with anti-Viperin antibody (Fig. 3b). There was no significant difference in the cell viability between PK-Vi, PK-C1 and PK-15 cells (Fig. 3c). In addition, as observed by confocal laser scanning microscopy, the expressed EGFP-Viperin protein distributed within the cytoplasm while EGFP distributed throughout the cell (Fig. 3d). These two cell lines were passaged for 10 generations and the expression of target proteins was confirmed by fluorescence microscopy and Western blot (data not shown), suggesting a stable gene expression.

### Over-expressing Viperin inhibited the replication of CSFV in PK-15 cells

**Fig. 4:**
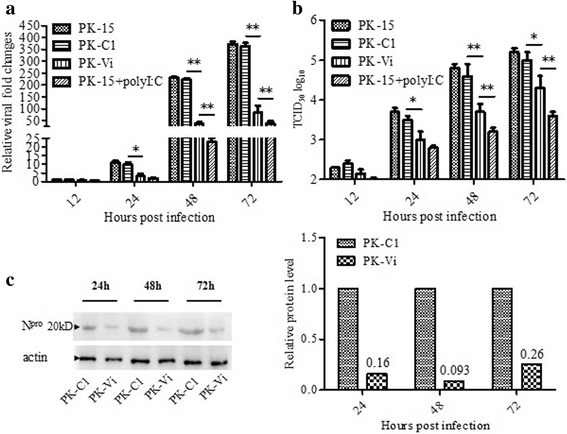
Over-expression of Viperin inhibits CSFV replication in PK-Vi cell line. **a** and **b**. CSFV genome RNA expression (**a**) and progeny virus (**b**) in PK-Vi was determined by qRT-PCR and virus titration. At 12, 24, 48 and 72 hpi, cell cultures were collected and lysed by three freeze and thaw cycles, then subjected to corresponding tests. **c.** Level of CSFV protein N^pro^ was determined by Western blot analysis. Cell lysates were harvested at 24, 48 and 72 hpi and detected with anti-N^pro^ antibody. An anti-β-actin antibody was used as an internal loading control. Protein spots levels were determined using ImageJ quantification software. Relative N^pro^ levels at each time point was that in PK-Vi group to that of PK-C1 group (referred as 1)

The inhibitory ability of Viperin on CSFV replication in PK-15 cells was determined by qRT-PCR and virus titration. Poly I:C treated and untreated PK-15 cells were used as negative and positive controls, respectively. As shown in Fig. 4a, at 24, 48, and 72 hpi, comparing to PK-C1 cells, the viral genome copy numbers in PK-Vi cell cultures were significantly decreased by 3.2-fold (*P* < 0.05), 6.1-fold (*P* < 0.01), and 4.4-fold (*P* < 0.01), respectively. In addition, comparing to PK-C1 cells, the titers of progeny virus in PK-Vi cells were significantly decreased by 3.2-fold (*P* < 0.05), 8-fold (*P* < 0.01) and 5.1-fold (*P* < 0.05) at 24, 48 and 72 hpi, respectively (Fig. 4b). In addition, virus in poly I:C treated PK-15 cells was dramatically decreased, which was significantly lower than that in PK-Vi cells at 48 and 72 hpi; while no significant difference was observed between PK-15 cells and PK-C1 cells (Fig. 4a, b). To further validate the results, Western blot analysis was performed using anti-N^pro^ antibody, N^pro^ expression levels decreased by 6.3-, 10.8- and 3.9-fold at 24, 48 and 72 hpi, respectively, due to the reduction of viral load (Fig. 4c). These results illustrated that the over-expressing Viperin inhibit the proliferation of CSFV.

### Viperin did not interfere with CSFV binding, entry and release in PK-15 cells

**Fig. 5:**
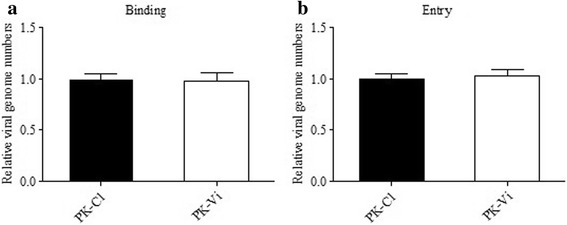
Detection of the effect of Viperin expression on CSFV binding and entry. **a**. Virus binding assay. Cells were inoculated with CSFV (MOI = 1) for 1 h on ice, washed with PBS. Cell-bound CSFV was measured by qRT-PCR. **b.** Entry assay. Virus inoculum was removed after 1 h of binding on ice, and then cells were washed with PBS and incubated in culture medium for 2 h at 37 °C. Cells were washed and trypsinated to remove bound but not entered virus before qRT-PCR detection. The relative genome levels of PK-Vi cells were normalized to those of PK-C1 cells (referred as 1). Data was shown as the mean ± S.D. from three independent experiments

**Fig. 6:**
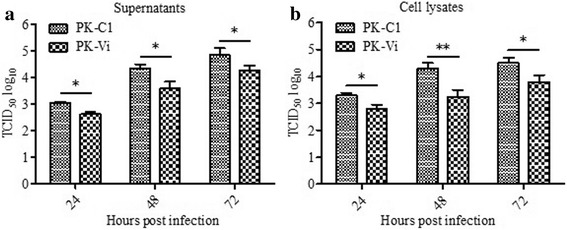
Detection of the effect of Viperin expression on CSFV release. **a** and **b**. CSFV was inoculated in PK-Vi and PK-C1, at 24, 48 and 72 hpi, cell culture supernatant (**a**) and cell pellet (**b**) were collected separately, and subjected to virus titration. Each test was performed in triplicate and data was shown as the mean ± S.D. Columns at each time point marked with * (*P* < 0.05) or ** (*P* < 0.01) are significantly different from each other

To test the effect of Viperin on virus binding and entry, experiments were performed according to previous report [27] and the CSFV genome copy numbers were determined by qRT-PCR. As shown in Fig. 5, no significant difference (*P* > 0.05) of the amount of binding virus was observed between PK-Vi and PK-C1 cells. Similarly, the amount of entry virus showed no obvious difference (*P* > 0.05) between the two cell lines. Furthermore, viral load in the cells and cell culture supernatants were determined. Compared with those of PK-C1 control cells, both of the viruses in PK-Vi cell culture supernatants and cell pellets were decreased with a similar proportion at 24, 48 and 72 hpi, respectively (*P* < 0.05, Fig. 6), imply that Viperin expression had no effect on viral release in PK-15 cells.

### Viperin co-localized with CSFV E2 and NS5B proteins and interacted with E2 protein

**Fig. 7:**
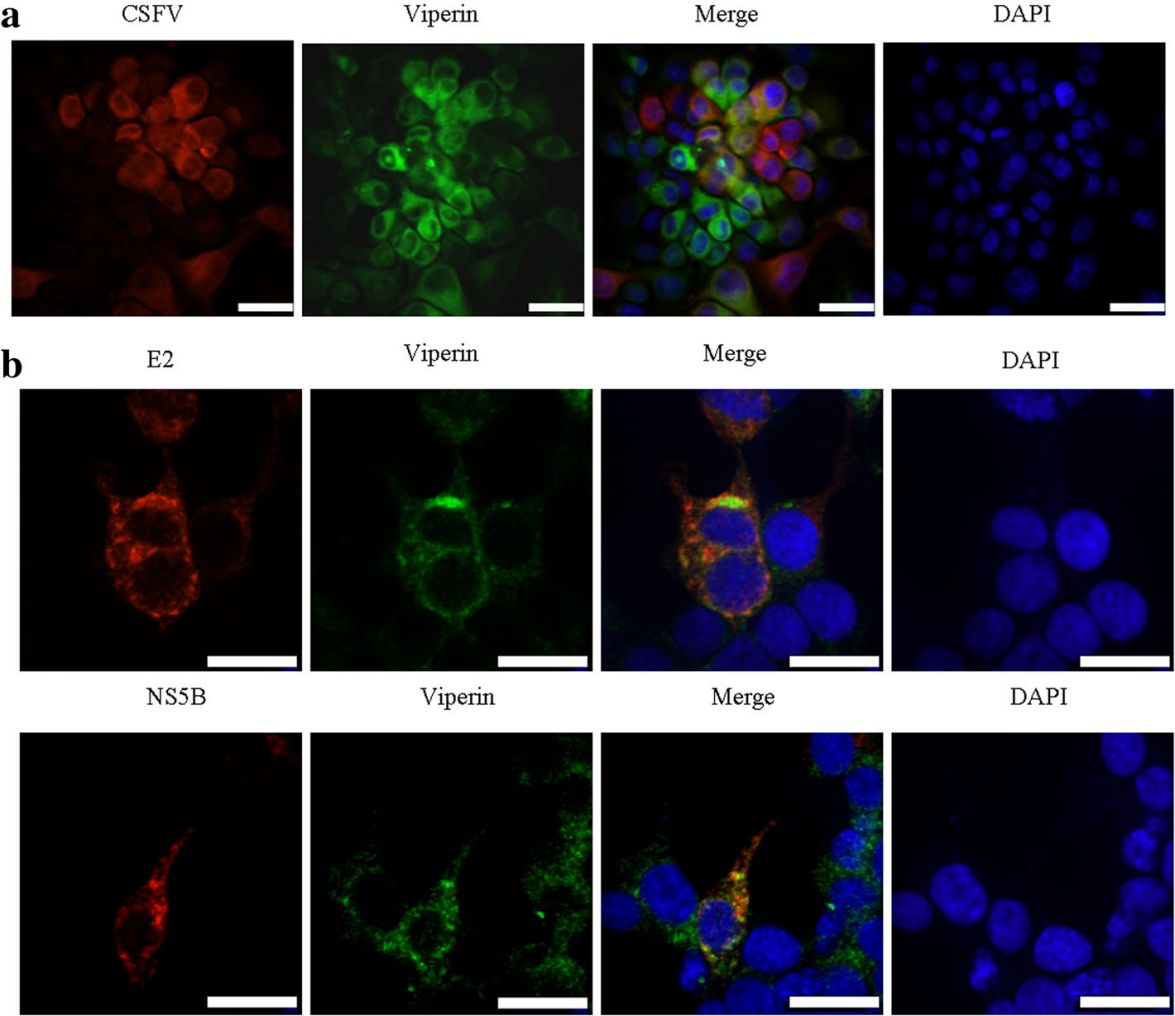
Confocal microscopy examination of Viperin with CSFV proteins. **a**. PK-Vi cells were infected with CSFV (MOI = 0.05) for 48 h, the cells were fixed with 4% paraformaldehyde, permeabilized and immune-stained with anti-E2 antibodies (WH303 mAb), followed by incubation with Cy3 (red)-conjugated goat anti-mouse secondary antibodies. Nuclei were stained with DAPI (Beyotime, Biotech, China) and the cells were examined by confocal microscopy (PE, Ultra View VOX). **b.** 293 T cells were co-transfected with pcDNA-Vi and pCMV-E2 or pCMV-NS5B and incubated at 37 °C for 48 h. Cells were fixed and immunolabelled for Viperin (rabbit anti-HA Ab) and E2/NS5B (anti-flag mAb) plus Alexa Fluor 488-labeled (green) goat anti-rabbit and Alexa Fluor 555-labeled (red) donkey anti-mouse secondary antibodies. Nuclei were stained with DAPI and examined via confocal microscopy (PE, Ultra View VOX). Scale bar indicated 20 μm

**Fig. 8:**
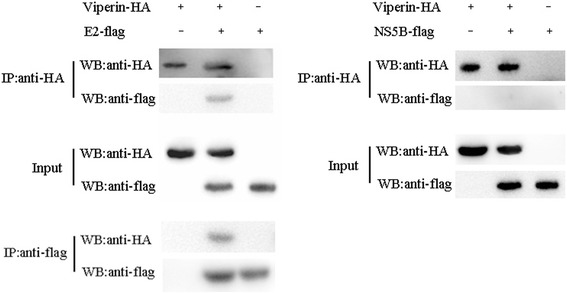
Interaction of Viperin with E2 and NS5B proteins was examined by co-immunoprecipitation (co-IP). 293 T cells plated in 6-well plates were transfected with pcDNA-Vi and pCMV-E2 or pCMV-NS5B. At 48 hpi, the cell lysates were immunoprecipitated by anti-HA or anti-flag antibodies and subjected to Western blot with rabbit anti-HA and anti-flag antibodies

Viperin expressing PK-Vi cells infected with CSFV were fixed, stained with CSFV specific antibody (WH303, target to E2) and then examined by confocal laser scanning microscopy. As shown in Fig. 7a, green fluorescence of EGFP-Viperin fusion protein overlapped with red fluorescence of CSFV in part of the cells, demonstrating the proximity of Viperin with CSFV proteins (i.e. E2) within the cell. We thus assessed the cellular localization of Viperin with E2 and NS5B proteins of CSFV in 293 T cells transfected with Viperin and E2 or NS5B expressing plasmids. As shown in Fig. 7b, Viperin and E2/NS5B signals were overlapping in a granular cytoplasmatic staining pattern. Furthermore, co-IP assay was performed to check the interaction of Viperin with E2/NS5B protein. As shown in Fig. 8, flag-tagged E2 protein was detected in immunoprecipitates obtained with anti-HA antibody; while flag-tagged NS5B protein was not detected after immunoprecipitation. In addition, HA-tagged Viperin could also be precipitated by flag-tagged E2. This indicated that Viperin interacted with the E2 protein.

## Discussion

Viperin is a newly identified ISG that has recently received increasing attention. It could be induced by type I (α and β), II (γ) and III (λ) IFNs, double stranded B-form DNA, poly I:C, lipopolysaccharide (LPS) and many viruses. The induction of Viperin is mediated by the classical IFN stimulated gene induction pathway and the IFN-independent pathway [5]. It appears to have a number of functions, from being an antiviral protein to modulating signaling events [28]. Recently, Viperin has been shown to have antiviral activity against many viruses, and many reports had shown Viperin expression could be highly induced by these viruses [5, 29–32]. In this study, the effect of CSFV infection on Viperin expression was studied in 3D4/21 cells and PBMCs, the results showed that CSFV infection does not significantly induce Viperin expression in both of the cells. Similarly, He et al. [22] had found CSFV could not induce Mx1 production in vitro. In the co-inoculate cells (NDV + CSFV; PRV + CSFV), Viperin mRNA expression induced by NDV/PRV was significantly reduced. Studies have showed CSFV infection could inhibit the production of type I IFN and inhibit poly(I:C) induced α and β IFN synthesis by the interaction of N^pro^with IRF3/IRF7 [19, 20, 33, 34]. The present results were consistent with previous reports. We speculated that the inhibitory effect of CSFV on Viperin was closely related to the innate immunity/IFN inhibitory characteristics of CSFV. In addition, CSFV could proliferate in co-infected cells (NDV + CSFV or PRV + CSFV) but the titers were lower than those of CSFV infected cells, suggesting the replication of CSFV was inhibited by NDV or PRV induced IFN responses. It also indicated the inhibitory effect of CSFV on Viperin mRNA expression was indeed caused by the replication of CSFV.

In China, CSF is still one of the most economically important diseases for the swine industry. National vaccination with C strain has been carried out for decades; however, infections with CSFV are still detectable and impair the pig industry [35, 36]. Several molecular biological techniques, antiviral drugs and proteins have been examined for anti-CSFV activity. Among them, Capsid-targeted virus inactivation, RNA interference, antiviral agents/ISGs such as Imidazole[4,5-c]pyridines, human MxA, porcine Mx1 and guanylate-binding protein 1 (GBP1) have been shown to inhibit CSFV replication in vitro [21, 37–40]. Several new anti-CSFV ISGs have been screened by using the reporter virus expressing Renilla luciferase (Rluc) [41]. In this study, Viperin over-expressing cell line was constructed to examine its antiviral activity, and the fusion expression of Viperin with EGFP facilitated the selection process. After CSFV inoculation, the highest viral reduction was observed at 48 hpi (Fig. 3). It was also true in Western blot test, Viral N^pro^ protein expression was highly reduced at 48 hpi (10.8-fold). These results demonstrated that Viperin effectively suppresses CSFV proliferation in both of the viral RNA and protein levels.

The mechanism by which Viperin restricts replication of different viruses has been studied, but it is still not fully understood. The initial characterization of Viperin revealed its antiviral ability on HCMV [8, 9]. Viperin likely exerted its anti-HCMV effects at a late stage of the viral life cycle, thus slowing the rate of transport of soluble proteins from the ER [42]. For influenza A virus, Viperin targeted lipid rafts to interfere virus release from the plasma membrane of infected cells [12]. In this study, the viral yield in cell culture supernatants and cell lysates showed an equally reduction with similar pattern, suggesting Viperin did not influence virus release. And virus binding/entry examination indicated the Viperin expression did not impair virus binding and entry. For Dengue virus type-2, Viperin inhibited viral RNA synthesis and co-localized with viral CA and NS3 proteins [30]. And in HCV, Viperin interacted with NS5A and the host factor VAP-A to limit virus replication [43]. In the present study, results from confocal test in CSFV-infected PK-Vi cells displayed Viperin co-localized with E2 protein. We speculated that the inhibition of CSFV replication by Viperin takes place in the cytoplasm by interaction of Viperin with CSFV proteins. To verify this hypothesis, the major structural protein E2 and nonstructural protein NS5B (crucial for virus replication) were selected for further examination. Co-localization between Viperin and E2/NS5B could be observed in the cytoplasm, although the fluorescence signal was not fully overlapped. Co-IP results confirmed the interaction of E2 protein with Viperin. Meanwhile, NS5B protein showed partially co-localization with Viperin, but the interaction with Viperin was not detected by co-IP. Studies had shown that the interaction of β-actin and Annexin 2 with E2 regulate the replication of CSFV [44, 45]. Thioredoxin 2 has also been reported as a novel E2-interacting protein that inhibits the replication of CSFV [46]. But the interacting partner of NS5B has not been reported [47]. We hypothesized that the antiviral activity of Viperin is potentially exerted through interaction with E2, which interfering the transport process of E2 and/or virion morphogenesis. Of course, the interaction of Viperin with other proteins (such as NS3 and NS5A) might also exist and crucial for its antiviral activity. These need to be identified in the future; and the crucial regions in Viperin responsible for anti-CSFV function will also be identified.

## Conclusion

The results presented in this study gave a profile about the interaction of CSFV infection and Viperin response; provided support that porcine Viperin could inhibit CSFV replication in vitro; the function occurred during the genome and/or protein synthesis step (not the entry or release step) and potentially via the interaction of Viperin with E2 protein. Our findings should be useful for CSFV-host interaction study in the future.

## Abbreviations

BDV: Border disease virus
BVDV: Bovine viral diarrhea virus
CSFV: Classical swine fever virus
HCMV: Human cytomegalovirus
IFN: Interferon
IRF: Interferon regulatory factor
ISGs: Interferon-stimulated genes
NDV: Newcastle disease virus
PBMC: Peripheral blood mononuclear cells
PRV: Pseudorabies virus
WNV: West Nile virus
